# Is It Training Enough? Professional Competence in Catering Quality for University Food Canteen Employees

**DOI:** 10.3390/foods13010059

**Published:** 2023-12-22

**Authors:** Yugang Ji, Wen-Hwa Ko

**Affiliations:** 1 Program in Nutrition and Food Science, Fu Jen Catholic University, New Taipei City 242062, Taiwan; 2 Guangdong University of Petrochemical Technology, Maoming 525011, China; 3 Department of Restaurant, Hotel and Institutional Management, Fu Jen Catholic University, New Taipei City 242062, Taiwan

**Keywords:** university canteen, food quality, professional competence, importance–performance analysis (IPA), employee education

## Abstract

The purpose of this study is to examine the relationship between professional competence and the training provided for canteen staff in Chinese universities. This study’s methods included a survey questionnaire, and importance–performance analysis (IPA) was used for analysis. The questionnaire distributed to canteen staff in Chinese universities considered eight dimensions and 39 questions to evaluate the relationship between the staff’s professional competence (performance level) and the training provided (importance level). “Focus on consumers”, “Employee hygiene knowledge”, and “Food quality” indicated poor professional competence for employees and insufficient training from the canteen. Our findings improve these circumstances by providing recommendations for future training. The research results provide guidance for managing and training university canteen employees and recommendations for improving the quality of catering.

## 1. Introduction

Catering quality is the core factor of restaurant value. Good catering quality is the key to successful restaurant operations [1]. In recent years, people have begun paying more attention to the quality of catering to ensure food safety. For example, many Chinese people are willing to pay a higher price for organic food because they believe in its safety and quality [2]. With the development of the market economy, Chinese colleges and universities began gradually implementing the socialization of logistics, independent management, self-responsibility for profit and loss, and independent accounting. Currently, school canteen owners are facing great pressure in competition and operations; to be oriented towards consumer demand, canteen operators must attach great importance to the quality of catering to attract more consumers to their canteen establishments. Students’ consumption concept changes with improved living standards. Students’ requirements for catering quality are constantly increasing. They are no longer satisfied with eating enough but also want to eat well. Students also have requirements regarding the type of food and the quality. Many Chinese university canteens still believe that they are not short of customers; they can also lack competitive consciousness or consideration for the tastes and requirements of foreign and ethnic minority students. As a result, some students find it difficult to eat in the canteens and choose off-campus social catering, resulting in the loss of consumers.

Brown et al. [3] indicated that it is essential to train canteen staff regarding food and beverage quality management and provide them with professional food and beverage management capacity to meet consumer expectations. Learning how to improve service efficiency in canteens, strengthening safety management, ensuring the health and safety of teachers and students, enhancing the quality of dining, and improving the satisfaction of teachers and students have become topics attracting much attention. This study adopted Ji and Ko’s [4] canteen Food Quality and Competence Scale (FQCS) to investigate canteen staff’s perspectives and abilities in Chinese colleges and universities. The importance–performance analysis (IPA) method was used to evaluate the relationship between the degree of staff ability to manage food and beverage quality and the adequacy of canteen training. This model can lead to a better understanding regarding the priority order of the items to be improved (i.e., the order of importance). Moreover, it can help determine which items of the FQCS should be strengthened in canteen training and which items should be strengthened by the staff. This study provides a reference for improving the service and management of Chinese college canteens. It promotes the continuous improvement of operational efficiency and lasting vitality of Chinese college canteens.

## 2. Literature Review

### 2.1. Food Quality

Sulek and Hensley [5] indicated three characteristics that determine the quality of food and beverage: food safety, food attractiveness, and food acceptability, including taste, appearance, texture, color, temperature, and quantity. Food and beverage quality is essential in determining customer satisfaction and loyalty. Catering quality includes food presentation, taste, menu variety, health, and freshness [6], factors which influence customer satisfaction and behavioral intention. The restaurant environment can reshape customer perception and directly impact customer satisfaction. Tangible and intangible elements inside and outside a restaurant, including temperature, light, smell, noise, atmosphere, and music, have been found to maintain a restaurant’s existing customer base and attract new customers [7].

### 2.2. Professional Competence in Catering

Competence refers to a person’s capability to perform duties, roles, and other needs at work to achieve the goal of effective performance [8]. Competence can be defined as the possession of knowledge, skills, and unique characteristics, including the proficient performance required by specific standards in various situations [9]. Competence can also be a collection of these attributes, along with the necessary knowledge, skills, attitudes, and abilities to effectively complete the work. Recent research suggests that competence can be defined as a set of individual behaviors that lead to superior results in an aspect of a job [10]. Competencies are divided into two categories, namely, visible and intangible abilities. Visible ability includes technical and non-technical skills, while intangible ability includes self-concept, personal quality, physical quality, and behavior; both types affect job performance and situational performance [11]. Integrating various knowledge and skills in professional cooking is key to career success [12].

Studies have found that chefs should have a basic knowledge and literacy of culture, aesthetics, technology, products, services, management, and creativity; these attributes have been collectively referred to as innovative cooking ability [13]. A professional chef must understand cooking techniques and the science behind food creation [14]. Zopiatis [15] listed technical competence as a key criterion for successful chefs. Technical competencies include specific culinary skills such as knowledge of food service management and the ability to develop menus and recipes. The leading professional competencies of chefs include cooking skills, specialized knowledge, communication skills, sales and marketing, and food concepts [16].

### 2.3. Hospitality Training

Training is necessary for any organization seeking to meet the demands of this competitive market, adapt to technological change, and meet human capital needs [17]. Chiaburu and Tekleab [18] used training as a method to develop employees’ skills, knowledge, and abilities, which can ultimately improve their job satisfaction and performance. There is a consensus that effective training reduces errors by improving employee capabilities [19]; training can improve employees’ service-related knowledge, skills, and abilities and enhance their sense of self-efficacy, work control, and job satisfaction. Service training is crucial for hotels and essential to human resource management [20]. Since top managers are typically more concerned about company profits, shareholders, and customers, they emphasize skill-based training outcomes to improve organizational performance [21]. However, HRM researchers pay more attention to the cognitive outcomes (i.e., self-efficacy) and emotional outcomes (i.e., job satisfaction) for trainees to develop human capital resources and thus improve organizational effectiveness [22]. Ubeda Garcia et al. [23] found that training directly affects performance and indirectly affects human resource outcomes (such as satisfaction and engagement).

## 3. Methodology

### 3.1. Sample and Data Collection

This study recruited cafeteria workers from Chinese universities as the research subjects. A questionnaire was completed through convenience sampling from November 2020 to January 2021 from more than 40 university canteens in northeast, central, and southern China. Among the universities surveyed, 1/3 have more than 30,000 students, 1/3 have between 10,000 and 30,000 students, and 1/3 have less than 10,000 students. In this study, employees who had worked for cafeterias in Chinese universities for more than half a year were selected as the research participants. A total of 400 questionnaires were distributed in the prediction stage, and 298 valid questionnaires were recovered. The final questionnaire was designed through project and factor analysis from the prediction stage. A total of 1302 questionnaires were distributed and 844 were successfully recovered, with a recovery rate of 64.8%.

### 3.2. Measurements

This questionnaire was divided into three parts. The first part was intended for Chinese college canteen staff to evaluate their FQCS for catering quality. The second part evaluated the adequacy of canteen functional training for canteen staff in Chinese colleges and universities. The third part included personal background information. The questionnaire for FQCS evaluation of dining quality in Chinese university canteens was developed using the scale from Ji and Ko [4]. The Cronbach’s alpha value of the entire scale is above 0.8, indicating good reliability. The scale was divided into eight dimensions, including kitchen safety (7 items), warehouse safety (5 items), food safety (5 items), staff hygiene knowledge (4 items), staff management (4 items), service awareness (3 items), attention to consumers (3 items), and food quality (8 items), with a total of 39 items. The five-point Likert scale was used to measure the degree to which employees rated their professional competence and the adequacy of the courses offered on a scale of 1 (very little/quite lacking) to 5 (very good/very sufficient).

### 3.3. Data Analysis

**Figure 1:**
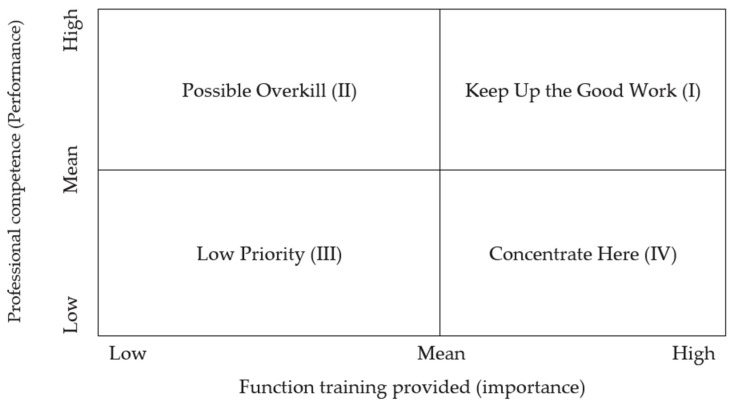
IPA diagram of professional competence by self-evaluation and functional training provided by Chinese universities’ canteens.

This study used the Statistical Package for Social Sciences (version 25.0) for data analysis. It also used the IPA analysis method to measure the relationship between the canteen staff’s catering quality ability and the functional training demand in Chinese universities. Factor coordinates were completed according to the mean value of individual catering quality, professional competence, and training provided. The graphical representation of the data produced four quadrants; the coordinates are divided into four quadrants for identification. Quadrant one indicates high professional competence and adequate training provided by the canteen (this means “Keep Up the Good Work”; it is sufficient for the training). Quadrant two indicates high professional competence and less training provided by the canteen (this means “Possible Overkill”; the organization needs to regularly confirm this competence). Quadrant three indicates poor professional competence and less training provided in canteens (“Low Priority”; it is necessary to strengthen training to enhance competence). Quadrant four indicates that professional competence is poor but the canteen provides adequate training (this means “Concentrate Here”; the training did not achieve the expected outcome and still failed to enhance professional competence) (Figure 1).

## 4. Results

This study distributed 1302 questionnaires to employees who had worked in the canteens of Chinese universities for more than half a year, and 844 valid questionnaires were recovered. The recovery rate was about 64.82%. The proportion of males was 47.04%, and females accounted for 52.96%. The proportion of participants aged 41 to 50 was 49.64%. The proportion of junior high school and below was 56.87%. Management positions accounted for 23.82%, and non-management positions accounted for 76.18%. Employees who had been working in a canteen for 1 to 4 years (inclusive) accounted for 21.68%, followed by 8 to 12 years (inclusive) accounting for 19.79%. Waiters accounted for 27.73%, followed by cooks accounting for 23.34%. Kitchen workers accounted for 18.36%; canteen administrators accounted for 9.48%.

### 4.1. Self-Evaluation of Catering Quality and Professional Competence of Canteen Staff in Chinese Universities

Table 1 shows the professional competence of canteen staff in Chinese colleges and universities classified into eight dimensions. Among them, the interviewed employees generally think that their professional competence is the highest in “Employee management”. “Service consciousness” and “Food safety” are second and third, respectively. Low scores for “Employee hygiene knowledge”, “Focus on consumers”, and “Food quality” indicated that employees were the least self-aware in these areas.

Table 2 shows items regarding the professional competence in catering quality of the canteen staff in Chinese universities. Among the 39 items, “B8. Employee Health Check Management (Should Have a health Certificate)” has the highest score. Next is “B6. Employee admission rules (washing hands and appearance, clothing and appearance, wearing masks and gloves, and talking as little as possible)” and “A6. Hygiene and safety management during the food (or catering) preparation process”. The lowest-score items were “D8. The Dishes are Innovative, and Rich in Variety” and “D5. Nutritional combination of Food”, indicating a low level of self-perceived professional competence in these areas.

### 4.2. Functional Training Provided by Chinese Universities’ Canteens for Employees

Table 3 shows the evaluation results regarding functional training adequacy provided by canteens while training staff in food and beverage quality management. In all eight dimensions, “Employee management”, “Service consciousness”, “Kitchen safety”, and “Food safety” were considered to include adequate training. This indicates that employees considered that this training helped them acquire F&B quality management skills. On the other hand, the courses on “Focus on consumers”, “Employee hygiene knowledge”, and “Food quality” were inadequate. Therefore, college canteens should pay more attention to providing these aspects of training.

Table 4 indicates the employees’ evaluation of whether the training of catering quality management competence provided for each question item is sufficient. Of those 39 entries, “B8. Employee health check management (should have a health certificate)”, “A23. Cleanliness of catering and serving areas”, and “C5. Pay attention to appearance and clothing” (e.g., work clothes and hats are neatly worn, clean, and hygienic, and “the ID badge is displayed as per the standard)” scored the highest. This was followed by “A18. Periodic elimination of pests and rodents in kitchens” and “A6. Hygiene and safety management during the food (or catering) preparation process”, “D3. Taste of food (or meal) (e.g., chewiness, crispness)”, “D8. The dishes are innovative and rich in variety”, “D5. Nutritional combination of food”, indicating that the training on these items is insufficient.

### 4.3. IPA Analysis of Professional Competence and Training Provided

**Figure 2:**
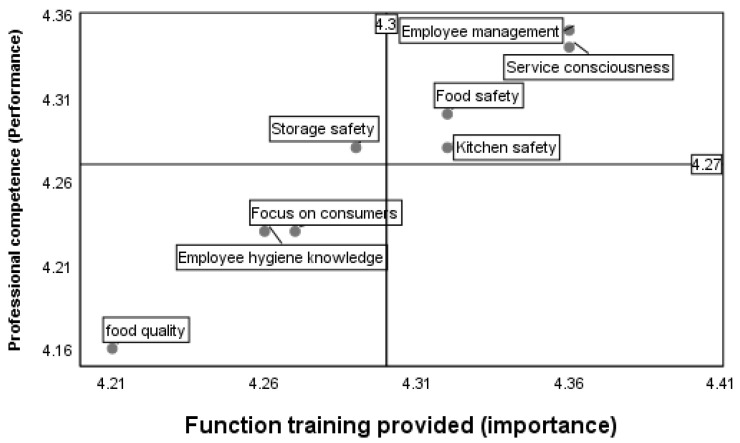
IPA analysis of the professional competence and training provided for the eight dimensions.

This study used IPA analysis to distribute all dimensions of catering quality and professional competence into four quadrants and analyze them separately in each quadrant. Figure 2 shows the significant differences in training and expertise across the eight dimensions. All dimensions were divided into four quadrants for further analysis. “Kitchen Safety”, “Food safety”, “Employee Management”, and “Service consciousness” were in the first quadrant of “Keep Up the Good Work”. This means that the training provided for these four dimensions was adequate; the performance was good. Storage Safety was in the second quadrant, “Possible Overkill”, i.e., the professional competence was considered high, but the training was insufficient, and the employees had professional competencies. However, canteen training could still be enhanced so that employees learn important concepts correctly. “Focus on consumers”, “Employee Hygiene knowledge”, and “Food quality” were in the third quadrant of “Low Priority”. This indicates that employees’ personal and professional competence in these three dimensions was poor, the canteen provided less training, and the performance was low. These results show that education and training must be strengthened in these areas.

**Figure 3:**
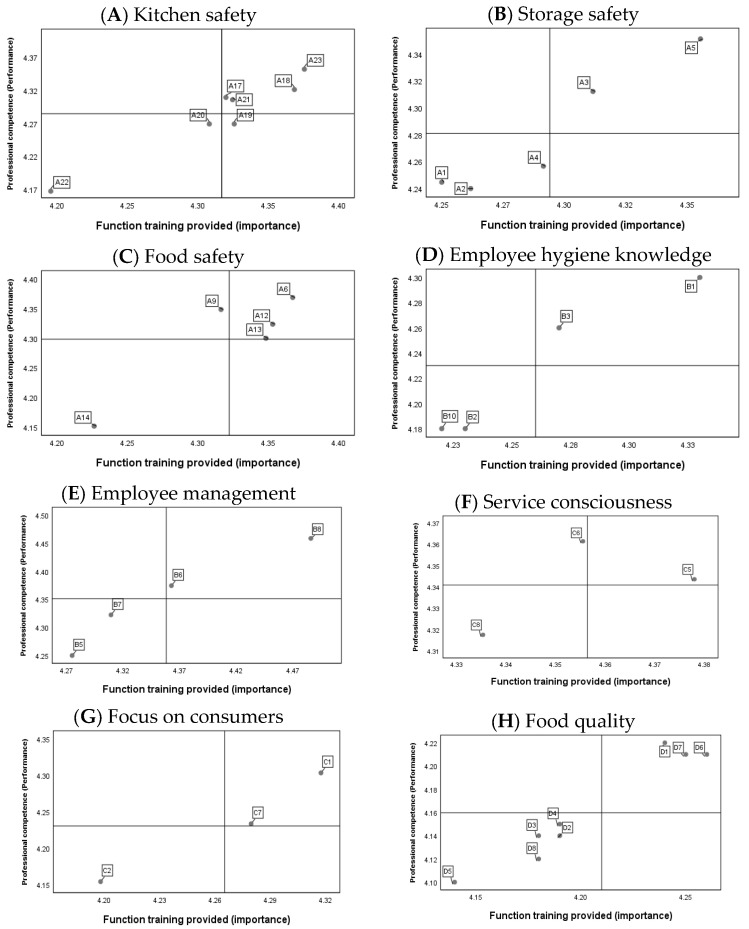
IPA analysis of the perceived training provided and perceived competence items for each dimension.

As shown in Figure 3, in the IPA of 39 individual items in eight dimensions, only “A19. Correct use and management of food Science” of “Kitchen safety” was in the fourth quadrant. This indicates that relevant functional training has been provided in the canteens of Chinese universities.

## 5. Discussion and Recommendations

### 5.1. Strengthen Employees’ Awareness of Quality and Hygiene Standardization

Restaurant food safety and hygiene are factors that consumers pay attention to when choosing where to eat their meals [24]. Foodborne diseases are generally caused by inadequate food preparation and storage facilities in food preparation sites that do not meet hygienic standards. School canteens should prioritize food safety and hygiene to avoid foodborne diseases [25]. The lack of food safety knowledge of canteen staff may lead to low awareness of food safety during food handling [26]. Employees must acquire food safety knowledge which can minimize outbreaks of foodborne diseases [27].

Employee behavior has a significant impact on pollution and may reduce the quality of the final product. University canteens should continue to provide staff health knowledge training. Canteens must master food hygiene and safety knowledge and food handling skills through training and constantly improve employees’ understanding of food (catering) safety and health, perceptions of food (catering) pollution prevention methods, equipment maintenance, and risk hazards. They also can standardize after-dinner services (for example, adding convenient facilities such as hand washing facilities), and strengthen the construction of the canteen system through training to familiarize employees with food safety laws and other relevant laws and regulations (such as food safety laws, HACCP, and GHP). Another recommendation involves the staff; through training certificates, it is recommended to improve the staff entry standards (hand washing and disinfection, clothing appearance, and not speaking). It is important to change the behavior and attitude of employees through training so that employees pay greater attention to the importance of hygiene and maintain high standards of personal and environmental hygiene to reduce the incidence of foodborne diseases and other health hazards.

The results of this study also show that staff have poor professional competence in “Food quality”, and canteens provide less training in this regard. According to the actual needs of consumers, the training in chefs’ cooking skills should be strengthened. Starting with the color, aroma, nutritional value, and freshness of dishes, canteens should pay attention to the collocation of food appearance with color, taste, and nutrition to improve the professional competence of chefs.

### 5.2. Enhance Employees’ Perception of Service

Kivela et al. [28] found that in the catering industry, customers pay attention to the quality of dishes but pay even more attention to the service quality during the dining process. Sometimes, customers think service quality is more important than the quality of the dishes. As an essential site for nutrition and health protection, the food provided by school canteens plays a key role in nutrition and safety [29]. The quality of a dish depends to a large extent on the skill of the person who cooks it. If the culinary staff emphasize the cooking process, they will carefully cook the dishes to achieve high quality [1].

Training content is positively correlated with the skills or cognitive outcomes of employees. The training process improves employees’ skills and abilities to address tasks more effectively [30]. Training has been a central part of high-performance human resource practices to help improve employees’ knowledge, attitudes, and work performance [31].

Service quality is an essential determinant of customer satisfaction and return visits. Improving service quality can help restaurant operators compete, retain old customers, and attract new customers. Paying attention to consumers is essential to improving service quality because employees’ service performance directly affects customer satisfaction. Therefore, this study suggests that canteen staff in colleges and universities should comprehensively enhance their service quality.

### 5.3. Management and Practical Implications

To strengthen the education and cultivation of staff’s work values, we can collaborate with the relevant departments of institutions to conduct training for staff regularly.

Canteen managers should strengthen the communication ability of the staff, let teachers and students perform restaurant hygiene and quality checks, and humbly accept the teachers’ and students’ opinions and suggestions. To strengthen canteen supervision, canteen management personnel can perform time-to-time spot checks on the quality of catering, the catering environment, and personal staff hygiene to ensure the quality and safety of university teacher and student dining. Furthermore, management should establish competition and reward and punishment mechanisms, stimulate chefs’ enthusiasm, and constantly improve the quality of dishes to enhance consumer satisfaction. The canteens should hire excellent chefs at high salaries to improve the quality of their restaurant dishes. Finally, school canteen chefs can be sent to participate in provincial and municipal cooking competitions or cooking training to improve their personal cooking ability.

### 5.4. Limitations and Future Studies

In this study, due to the particularity of college canteens in China, there are considerable differences in the size and quantity of college canteens, the overall quality of employees, and the funds and training efforts invested by schools. Future studies can analyze the differences and connections between college canteens at different levels.

This study’s subjects were canteen employees in Chinese universities. Cross-sectional comparisons can be made between hotel industry employees in future studies to determine the consistency of results among different groups. Future research may consider expanding the sample of colleges and universities involved to further explore the relationship between the individual competence of canteen staff and functional training.

## Figures and Tables

**Table 1:** Mean and SD for dimensions of professional competencies in catering quality management for Chinese universities’ canteen employees.

Dimension	Mean	SD	Ranking
Kitchen safety	4.28	0.588	4
Storage safety	4.28	0.608	4
Food safety	4.30	0.590	3
Employee hygiene knowledge	4.23	0.637	6
Employee management	4.35	0.589	1
Service consciousness	4.34	0.585	2
Focus on consumers	4.23	0.639	6
Food quality	4.16	0.630	8
Total	4.27	0.540	

Note: *n* = 844.

**Table 2:** Mean and SD for items of professional competencies in catering quality management for Chinese universities’ canteen employees.

Dimension	Items	Mean	SD	Ranking of Dimension	Total Ranking
Kitchen safety	A17. Proper use and control of detergents and disinfectants	4.31	0.675	3	13
	A18. Periodic elimination of pests and rodents in kitchens	4.32	0.704	2	9
	A19. Correct use and management of food additives	4.27	0.743	5	19
	A20. Waste (e.g., food waste and expired products) treatment and removal methods	4.27	0.721	5	19
	A21. Covering trash bins and sorting garbage	4.31	0.683	3	13
	A22. Air quality in the kitchen	4.17	0.757	7	32
	A23. Cleanliness of catering and serving areas	4.35	0.665	1	5
Storage safety	A1. Traceability of the sources of food materials	4.24	0.777	4	24
	A2. Food material inspection and quarantine certificate	4.24	0.802	4	24
	A3. Compliance with the relevant regulations for storage hygiene management (classification, storage on separate shelves, and registration of materials)	4.31	0.716	2	13
	A4. Time and temperature control during food storage	4.25	0.727	3	22
	A5. Food material management follows the first-in, first-out warehousing principle	4.35	0.711	1	5
Food safety	A6. Hygiene and safety management during the food (or catering) preparation process	4.37	0.693	1	2
	A9. The clean and hygienic environment in the kitchen	4.35	0.669	2	5
	A12. Proper handling of overnight meals according to standards	4.32	0.705	3	9
	A13. Cautious attention to possible cross-contamination in the food environment (such as contamination of raw and cooked foods, and peeling of wall coverings)	4.30	0.703	4	16
	A14. Periodic safety inspection of drinking water and ice cube hygiene	4.15	0.741	5	33
Employee hygiene knowledge	B1. Employees’ awareness of food (or catering) safety and hygiene	4.30	0.686	1	16
	B2. Employees’ awareness of food (or catering) contamination prevention methods	4.18	0.719	3	30
	B3. Employees’ awareness of food (or catering) risks and hazards	4.26	0.705	2	21
	B10. Chefs and kitchen helpers have basic knowledge of cooking nutrition	4.18	0.778	3	30
Employee management	B5. Employees are familiar with food (or catering) safety and sanitation operating specifications	4.25	0.709	4	22
	B6. Employee admission rules (washing hands and disinfection, clothing and appearance, wearing masks and gloves, and talking as little as possible)	4.37	0.664	2	2
	B7. Mandatory employee independent hygiene management	4.32	0.686	3	9
	B8. Employee health check management (should have a health certificate)	4.46	0.637	1	1
Service consciousness	C5. Pay attention to appearance and clothing (e.g., work clothes and hats are neatly worn, clean, and hygienic, and the ID badge is displayed as per the standard)	4.34	0.645	2	8
	C6. Service attitude (e.g., civilized language, kind attitude, polite to others)	4.36	0.660	1	4
	C8. Respect the privacy of consumers (including students)	4.32	0.654	3	9
Focus on consumers	C1. Attach importance to communicating with consumers (including students) and listening to opinions	4.30	0.721	1	16
	C2. Understand catering expertise	4.15	0.732	3	33
	C7. Responding to and handling complaints from consumers (including students)	4.23	0.712	2	26
Food quality	D1. Food (or meal) appearance and color	4.22	0.709	1	27
	D2. The aroma of food (or meal)	4.14	0.730	5	36
	D3. Taste of food (or meal) (e.g., chewiness, crispness)	4.14	0.730	5	36
	D4. Food (or meal) is delicious	4.15	0.719	4	33
	D5. Nutritional combination of food	4.10	0.752	8	39
	D6. The temperature of food (or meal) served	4.21	0.702	2	28
	D7. Reasonable price of food (or meal)	4.21	0.707	2	28
	D8. The dishes are innovative and rich in variety	4.12	0.753	7	38

**Table 3:** Mean and SD for the dimensions of adequacy of functional training provided in catering quality management competence for Chinese universities’ canteen employees.

Dimension	Mean	SD	Ranking
Kitchen safety	4.32	0.610	3
Storage safety	4.29	0.628	5
Food safety	4.32	0.612	3
Employee hygiene knowledge	4.26	0.661	7
Employee management	4.36	0.614	1
Service consciousness	4.36	0.629	1
Focus on consumers	4.27	0.682	6
Food quality	4.21	0.657	8
Total	4.30	0.580	

Note: *n* = 844.

**Table 4:** Mean and SD for items of adequacy of functional training provided in catering quality management competence for Chinese universities’ canteen employees.

Dimension	Items	Mean	SD	Ranking of Dimension	Total Ranking
Kitchen safety	A17. Proper use and control of detergents and disinfectants	4.32	0.695	4	14
	A18. Periodic elimination of pests and rodents in kitchens	4.37	0.694	2	4
	A19. Correct use and management of food additives	4.33	0.700	3	12
	A20. Waste (e.g., food waste and expired products) treatment and removal methods	4.31	0.711	6	18
	A21. Covering trash bins and sorting garbage	4.32	0.712	4	14
	A22. Air quality in the kitchen	4.20	0.789	7	33
	A23. Cleanliness of catering and serving areas	4.38	0.684	1	2
Storage safety	A1. Traceability of the sources of food materials	4.25	0.767	5	27
	A2. Food material inspection and quarantine certificate	4.26	0.763	4	25
	A3. Compliance with the relevant regulations for storage hygiene management (classification, storage on separate shelves, and registration of materials)	4.31	0.719	2	18
	A4. Time and temperature control during food storage	4.29	0.723	3	21
	A5. Food material management follows the first-in, first-out warehousing principle	4.35	0.718	1	8
Food safety	A6. Hygiene and safety management during the food (or catering) preparation process	4.37	0.687	1	4
	A9. The clean and hygienic environment in the kitchen	4.32	0.703	4	14
	A12. Proper handling of overnight meals according to standards	4.35	0.684	2	8
	A13. Cautious attention to possible cross-contamination in the food environment (such as contamination of raw and cooked foods, and peeling of wall coverings)	4.35	0.702	2	8
	A14. Periodic safety inspection of drinking water and ice cube hygiene	4.23	0.758	5	30
Employee hygiene knowledge	B1. Employees’ awareness of food (or catering) safety and hygiene	4.33	0.713	1	12
	B2. Employees’ awareness of food (or catering) contamination prevention methods	4.23	0.755	3	30
	B3. Employees’ awareness of food (or catering) risks and hazards	4.27	0.736	2	23
	B10. Chefs and kitchen helpers have basic knowledge of cooking nutrition	4.22	0.778	4	32
Employee management	B5. Employees are familiar with food (or catering) safety and sanitation operating specifications	4.27	0.713	4	23
	B6. Employee admission rules (washing hands and disinfection, clothing and appearance, wearing masks and gloves, and talking as little as possible)	4.36	0.686	2	6
	B7. Mandatory employees’ independent hygiene management	4.31	0.709	3	18
	B8. Employee health check management (should have a health certificate)	4.49	0.658	1	1
Service consciousness	C5. Pay attention to appearance and clothing (e.g., work clothes and hats are neatly worn, clean, and hygienic, and the ID badge is displayed as per the standard)	4.38	0.663	1	2
	C6. Service attitude (e.g., civilized language, kind attitude, polite to others)	4.36	0.692	2	6
	C8. Respect the privacy of consumers (including students)	4.34	0.713	3	11
Focus on consumers	C1. Attach importance to communicating with consumers (including students) and listening to opinions	4.32	0.743	1	14
	C2. Understand catering expertise	4.20	0.773	3	33
	C7. Responding to and handling complaints from consumers (including students)	4.28	0.745	2	22
Food quality	D1. Food (or meal) appearance and color	4.24	0.748	3	29
	D2. The aroma of food (or meal)	4.19	0.744	4	35
	D3. Taste of food (or meal) (e.g., chewiness, crispness)	4.18	0.751	6	37
	D4. Food (or meal) is delicious	4.19	0.761	4	35
	D5. Nutritional combination of food	4.14	0.790	8	39
	D6. The temperature of food (or meal) served	4.26	0.721	1	25
	D7. Reasonable price of food (or meal)	4.25	0.720	2	27
	D8. The dishes are innovative and rich in variety	4.18	0.768	6	37

## Data Availability

Data is contained within the article.
